# The Effect of Telling Lies on Belief in the Truth

**DOI:** 10.5964/ejop.v13i4.1422

**Published:** 2017-11-30

**Authors:** Danielle Polage

**Affiliations:** a Central Washington University, Ellensburg, WA, USA; Webster University Geneva, Geneva, Switzerland

**Keywords:** lying, lies, inflation, memory, deception

## Abstract

The current study looks at the effect of telling lies, in contrast to simply planning lies, on participants’ belief in the truth. Participants planned and told a lie, planned to tell a lie but didn’t tell it, told an unplanned lie, or neither planned nor told a lie (control) about events that did not actually happen to them. Participants attempted to convince researchers that all of the stories told were true. Results show that telling a lie plays a more important role in inflating belief scores than simply preparing the script of a lie. Cognitive dissonance may lead to motivated forgetting of information that does not align with the lie. This research suggests that telling lies may lead to confusion as to the veracity of the lie leading to inflated belief scores.

Garry, Manning, Loftus, and Sherman (1996) showed that participants who initially reported that certain childhood events had not happened, but then imagined they had happened, increased their confidence in the false events. This now famous effect is commonly referred to as the ‘imagination inflation’ effect, because it has been shown that through imagining an event their belief in the event becomes more likely, or their belief in the event becomes inflated. Since then, other studies have shown that imagination is not the only way to increase belief in counter-factual events. Studies have demonstrated an inflation effect by exposing participants to the false information using a variety of paradigms such as paraphrasing (Sharman, Garry, & Beuke, 2004) and explanations (Sharman, Manning, & Garry, 2005). Studies have also shown that making up information, or what researchers call “confabulating” information, results in memory failure for the truth. In Chrobak and Zaragoza (2008), participants were asked to describe entire fictitious events that they had never witnessed. Results show that over time, half of the participants developed false memories of these fictitious events. Pickel (2004) showed that participants who fabricated descriptions of a videotaped robbery suspect had trouble remembering the actual suspect and also confused their made up details with the truth. It appears that almost any task that increases the familiarity with and requires participants to mentally engage with the false information might lead to an inflation of belief effect. Many studies have looked at the effect on memory of creating false information; however, few studies have looked at the effect of intentional lying on memory for the truth. Polage (2012) demonstrated that deliberate lying can lead to “fabrication inflation” in which participants increase their likelihood ratings for self-created events after lying about them.

It is very likely that liars use their imagination to create false stories, but lying is more than just creating a false story. Dictionary.com defines a lie as “a false statement made with deliberate intent to deceive” (“Lie,” 2017). This definition involves two components: the first involves the veracity of the information and the second involves the deceptive intent of the speaker. Therefore, in order for a person to tell a lie, there must be evidence of both components of the definition. Liars must first construct false stories and then try to convince others that their stories are true. The current research examines whether the deliberate attempt to deceive plays a role in belief inflation or whether lying is simply another means of exposing participants to false information. To the author’s knowledge, there is no literature looking at the effect of telling, versus simply creating, a lie on likelihood to believe.

According to the Source or Reality Monitoring (RM) approach, source confusion occurs when people create perceptual and sensory details of false events that are similar to real memories (Johnson & Raye, 1981). When participants answer questions about the false event, whether in writing or speaking, the memory for the false event becomes more detailed and more similar to true event memories increasing the likelihood that the person will believe it. The exposure will also increase familiarity and fluency with the memory which is also thought to increase belief in the false memory (Garry & Wade, 2005). According to Vrij, Granhag, and Mann (2010), good liars look to the listener to determine whether they feel they are being believed and try to tell simple, plausible, and realistic lies that would not contradict anything the observer might know. Implausible events are unlikely to be believed (Pezdek, Finger, & Hodge, 1997), but memories of detailed, plausible events from one’s past might be confused with true events leading to an inflation effect.

If lying was simply another means of exposure to false information resulting in source confusion, then whether the lie was told would make no difference. But lies, unlike imagination, paraphrasing, guessing, and confabulating, must be communicated with the intention to deceive. So, what effect does the telling of a lie have on belief? Previous studies suggest contradictory outcomes. According to the Source Monitoring framework memories rich in detail might be considered true in the absence of memory for the cognitive operations used to create those memories. But, telling lies is effortful. Langleben et al. (2002) observed that the brain is more active during lie telling than it is while telling the truth. Zuckerman, DePaulo, and Rosenthal (1981) suggest that liars must maintain internal consistency (such as avoiding contradicting oneself) and external consistency (making sure the lies don’t contradict what others know to be true) and therefore lying requires more cognitive effort than telling the truth. One would expect this cognitively demanding task to be remembered, so even though the content of the memory might be remembered, so would the act of creating the lie. Any familiarity with the content of the lie could be correctly attributed to lying. Telling a lie without pre-planning would be especially cognitively demanding. Walczyk, Mahoney, Doverspike, and Griffith-Ross (2009) and Greene, O’Hair, Cody, and Yen (1985) point out that it takes less time to deliver a prepared lie than it does to tell the truth as the response is simply the delivery of a memorized script. DePaulo et al.’s (2003) meta-analysis reported that less time to plan a lie is related to greater cognitive effort. Regurgitating planned lies is less cognitively demanding than creating and telling lies on the spot. In terms of memory for cognitive processing, the increase in cognitive demand for the told lies should serve as a cue to distinguish between false and true memories and result in a decrease in belief in the lie. If memory for cognitive operations significantly impacts source monitoring, one would expect the told only group to show a lower inflation effect than told lies that were prepared first.

Although telling lies is cognitively demanding, several lines of research suggest that other aspects of telling a lie might increase source monitoring errors and lead to higher rates of fabrication inflation. Polage (2012) found that participants who reported feeling more uncomfortable about lying were more likely to believe their self-generated lies. It is possible that the negative affect involved in lying induces dissonance, which later becomes self-deception. Cognitive dissonance (Festinger, 1957) arises when one’s beliefs and actions contradict each other. An easy way to avoid the discomfort of having lied, is to believe that the lie is the truth. Shu, Gino, and Bazerman (2011) demonstrated how cognitive dissonance can result in memory change. Their study found that participants who first read an honor code and later cheated were less likely to remember the components of the honor code than were those who didn’t cheat. They suggest their results are due to motivated forgetting of the rules in an attempt to preserve one’s moral self-image after behaving unethically. Kouchaki and Gino (2016) also found that after engaging in unethical behavior, participants’ memories for their past unethical actions were impaired. The authors believe that the psychological distress and discomfort of their misdeeds caused the memories to become less clear and vivid than memories of ethical actions. They also suggest that this “unethical amnesia” could lead people to repeat acts of dishonesty. Polage found that those who lied frequently were much more likely to believe their lies than those who lied less often. It is possible that people revise their memories to reduce cognitive dissonance. Participants who find themselves uncertain as to the true source of the lied-about information and who are also motivated to want to believe the lie, may lower their decision criterion required to accept the lie as true (Hekkanen & McEvoy, 2002) and if successful this strategy may be repeated.

Another important difference between simply planning a lie and telling a lie is that until a lie is communicated to someone else, the liars are free to continually update and change their versions of their stories. A lie becomes a lie when it is shared. The goal of lying is to be believed. Good liars track the reactions of their audience and modify their stories and behavior to maintain the lie (Buller & Burgoon, 1996; Vrij et al., 2010). Once shared, liars should “stick with” their lie in order to avoid detection. So, although both planning and telling lies could provide the content of the lie, sharing the lie provides the motivation to remember the lie exactly as it was told in order to avoid detection. Wade, Garry, Nash, and Harper (2010) showed that false memories are affected by an “anchoring effect” in that the first version of the false event is most influential in memory distortion. The version of the story they created first became “truth” to them. Similarly, the first version of the event told should anchor the memory; the liar must then remember the details of the told lies to maintain the falsehood. The lie that was shared is now public and the liar could be motivated to commit it to memory, believe it, and accept it as collective “truth”.

So although the source monitoring literature might suggest that awareness of cognitive operations while telling a lie might lead to less inflation, studies suggesting that discomfort, lax criterion, motivated forgetting, cognitive dissonance, and maintenance of consistency could lead to higher levels of source confusion when a lie is shared.

Finally, it is possible that the act of simply speaking the lie out loud while trying to convince the other person they are telling the truth might help liars remember the content of the lie. Hopkins and Edwards (1972) showed that memory for words that were pronounced was better than for words that were studied silently. Therefore, speaking some lies out loud versus simply thinking about others might make the unspoken lies less memorable than those that were spoken. Saying the information out loud can increase memory for that information which would suggest that the content of the spoken lies will be more memorable.

In summary, fabricating a lie likely uses many techniques such as imagination and counterfactual thinking that have been shown to result in inflated belief in the false event. Lying differs however, in that lies are told to another person with the intention of deceiving the listener. Both planning and telling lies will result in created content for the lie which could inflate belief in the created content. However, in terms of ability to source monitor, telling a lie is thought to be more effortful and the cognitive effort could be remembered, especially in the absence of pre-planning the lie when the cognitive effort is greatest. If the creation of the lie is remembered, then familiarity with the content of the lie can be attributed to self-generation of the lie and belief in the lie should decrease. However, if cognitive dissonance is induced, motivated forgetting of having lied could counteract memory for cognitive operations and increase the likelihood of the lie being accepted as true. Lying to another person cements the lie as public truth providing the liar motivation to remember the lie and want to believe it. In the absence of clear counter memories, the liar may be more likely to accept the lie as truth and inflate their belief in the lie. Told lies might be more simple and plausible which could further increase the likelihood of believing the lie. Finally, telling a lie also benefits from the effects of speaking information aloud which has been shown to improve memory for the content of the lie and could make the source judgment more difficult. It is therefore expected that both planning and telling a lie should provide the content of a lie, but that telling a lie could lead to stronger impairments in ability to source monitor due to discomfort, motivated forgetting, and lax criterion. Telling a prepared lie should result in all of the impairments of planning and telling the lies such as rehearsal of the lie, cognitive dissonance, motivated forgetting and belief in public record, but in addition should result in an even more difficult source monitoring decision than either planning or telling alone due to repetition of the memory and decreased memory for cognitive operations (due to the decreased effort needed to tell a pre-planned lie). It is therefore expected that planning and telling a lie will have an additive effect and should result in the highest inflation effect. The current study compares the effects of planning and telling lies on belief in the lie and anticipates main effects for both planning and telling a lie, with the highest fabrication occuring for lies that are both planned and told.

## Method

### Participants

Fifty-two undergraduate students from Central Washington University participated in the first session of the study, four did not return for the second session, and data from three participants was discarded due to participants repeatedly not following the lying prompts. The resulting 45 participants were used in the final analyses.

### Materials

Participants' experiences were measured using the Life Events Inventory (LEI; Garry et al., 1996). The full inventory contains 60 items that ask whether a particular event happened to the participant before the age of 10. The participant rated whether each event happened using a Likert-type scale anchored at 1 (definitely did not happen) to 8 (definitely did happen). The LEI was administered twice, approximately 2 weeks apart. The pretest consisted of 21 of the 60 items from the original. Pretest responses were used to get baseline ratings of four target items to be used in the study. The ideal score for each of the four target items was a score of 2 which indicated participants were quite confident the events in question had not happened, however, the placement of the score on the scale still allowed for movement in both directions and avoided a floor effect. After selecting items with an initial rating score of 2, scores of 3 were utilized, then 1 and, finally, as a last resort, an item with a score of 4 was chosen. After selecting the four events, they were randomly assigned to one of the four event conditions (Lie 1, Lie 2, Lie 3, or Lie 4 described later). The posttest scores were used in order to compute a change score from pre-test to posttest. The posttest consisted of 42 items (the original 21 items plus 21 new items that served as distractors).

Participants were also given the Dissociative Experiences Scale (DES; Bernstein & Putnam, 1986) with the intent of using it as a covariate. The DES consists of 27 items that ask the participant to estimate how often an experience happens to them. For example, one question states, "Some people have the experience of driving a car and suddenly realizing that they don't remember what has happened during all or part of the trip." The participant is asked to estimate what percentage of the time this happens to them. Because its relationship to the dependent variables was not significant, it will not be discussed further in this paper.

### Design

The study was a within subjects repeated measures design with two independent variables. The first independent variable was whether the lie event was planned (or not) and the second independent variable was whether the lie event was told (or not). The dependent variable was the change in ratings score from LEI 1 to LEI2 taken two weeks later.

### Procedure

After receiving informed consent, participants were given the LEI to complete. After completing the LEI, participants were given instructions to read while the experimenter excused herself to use the bathroom. In reality, the researcher went into a neighboring room in order to score the LEI pretest and select which four life events would be used in the experiment. The experimenter randomly assigned the four events to be either prepared and told (Lie 1), not prepared but told (Lie 2), prepared but not told (Lie 3), or not prepared and not told (control).

The instructions provided to the participants included a cover story that explained that the study was designed in order to determine whether it was possible to tell whether someone is lying. They were told they would be asked about some events that may or may not have happened to them and that the interviewer would try to determine whether they were lying. The instructions further explained that the participants would be given items to describe to the interviewer and that they would be told whether to say that the event did or did not occur before the age of 10. So, whether the event happened or not, the participant was to follow the prompting of the interviewer. Participants were told that they should be as sincere as possible as the idea was to convince the interviewer that the event actually happened. If the event actually happened before the age of 10 and they were told to say that it happened, they were asked to include factual information. If the event did not occur before the age of 10 and they were told to say that it did happen, they were asked to tell a feasible story in order to convince the interviewer that the event actually happened.

When the experimenter finished scoring the LEI, she returned to the room and read the instructions with the participant to ensure they were understood. Then, she gave the participant an eight-item "events list" that the participant was asked to write stories about; this was the "prepared" manipulation. The events are listed in the Appendix in the order given to the participants. Note that the two lies come from the participants' LEI pretest scores and were randomly chosen from the four items that received a 2 (3, 1, or 4) score. The same six filler items were used for all participants and were not selected based on the ratings given on the first LEI; two “yes” responses which were likely to be true for most participants and four “no” responses, two of which were likely to be false and two of which were likely to be true for most participants. For each event on the list that participants would claim to be true, they were asked to answer eight follow-up questions: (1) What were you doing right before this event occurred? (2) Where were you? (3) Who were you with? (4) How old were you? (5) What time of day was it? (6) How did you feel about this event? (7) What happened right after the event? (8) Are there any other details important to this story? For the “no” responses, participants were asked to answer "How do you know that you never _______?". The “no” responses were not of interest to this study, but served as a counterbalance measure to avoid all “yes” responses. Participants were given as much time as they needed to complete this part of the experiment, and they generally finished in about 20-30 minutes. When they finished writing about the various events (i.e., "preparing" their lies), they summoned the experimenter from the adjoining room in order to do the next part of the study: the oral interview.

For the interview (the "told" manipulation), participants were asked to discuss a variety of events including Lie 1 (the same event they "prepared") and Lie 2 from the LEI pretest (which was not prepared but had to be created on the spot). For each event, participants were either asked to "Please tell me about the time that you __________" or to tell the interviewer "How do you know that you never _______?". The instructions were the same in the oral interview as for the written /prepared portion. They were directed to answer according to the interviewer’s prompt even if their response was not true. The same eight follow-up questions used in the writing session were used as prompts in the interview in the same order. The interview session also took about 20-30 minutes. After the interview was completed, they were reminded to return in two weeks for the second session.

At the second session, approximately 2 weeks later, participants were run singly or in small groups. They were asked to complete the LEI posttest which consisted of 42 events, repeating all 21 from the pretest in addition to 21 previously unpresented events from the original LEI. They were then provided with complete disclosure.

## Results and Discussion

The LEI pretest scores were subtracted from posttest scores on the four target events. Therefore, there were four change scores per participant: Lie 1 (prepared and told), Lie 2 (not prepared but told), Lie 3 (prepared but not told), and control (neither prepared nor told). If participants increased in their belief in the lie, their change scores should be positive. The mean (standard deviation) change scores are presented in Table 1 (below).

**Table 1:** Mean (SD) Change Scores for Experiment 1

Target Event	*M* (*SD*)
prepared and told (Lie 1)	1.51_ab_ (2.21)
not prepared but told (Lie 2)	1.02 (2.39)
prepared and not told (Lie 3)	0.80_a_ (2.08)
not prepared and not told (Lie 4: control)	0.44_b_ (1.98)

*Note*. Shared subscripts indicate a significant difference at *p* < .008.

Telling a lie about an event (*M* = 1.27) did increase the belief that the event did occur relative to not telling a lie (*M* = 0.62; *F*(1, 44) = 5.05, *p* = .03, η^2^ = .103). There was no main effect for preparation of the lie (*F*(1, 44) = 1.66, *p* = .20, η^2^ = .036). There was no significant interaction between preparing the event and lying about it (*F*(1, 44) = 0.06, *p* = .81, η^2^ = .001). Planned within subject t-test comparisons were conducted using a protected alpha level to test the hypothesis that planning combined with telling would increase inflation effects as compared to simply telling or planning alone. Results demonstrated that the planned and told group caused significantly higher inflation scores than the planned only group. There was no significant difference between planning or not planning told lies (See Table 1). The results demonstrate that telling a lie to another person in an effort to deceive, and not simply creating a lie, increases belief in the lied about event. Telling lies, whether planned or not, resulted in the highest change scores.

These results support previous results (Polage, 2012) demonstrating that telling lies is yet another paradigm in the long list of methods used to inflate belief in false, self-generated information (imagining: Garry et al., 1996; paraphrasing: Sharman et al., 2004; explanations: Sharman et al., 2005; confabulating: Chrobak & Zaragoza, 2008; Pickel, 2004). It is likely that constructing a lie uses many similar processes as other inflation tasks which result in detailed memories and increased fluency with the memory; however, the current results suggest that it is the telling of the lie and not just the creation of the lie that drives fabrication inflation. As Vrij et al. (2010) suggests, good liars tell simple, plausible, and realistic lies which should be easier to believe than complicated, unrealistic lies. Attempting to be believable constrains realism that would not be present when simply imagining an event. So, although forming a lie may involve imagery, it may also differ from simple imagination in which there is no external pressure to be believable. Lying also cements the lie as public truth providing the liar motivation to remember the lie and want to believe it. In the absence of clear counter memories, the liar may be more likely to accept the lie as truth and inflate their belief in the lie.

But telling a lie is thought to be effortful (Langleben et al., 2002; Zuckerman et al., 1981) which should result in the correct attribution of even a clear and realistic memory as having been fabricated. So why would the told lies show higher inflation than lies that weren’t told? It was expected that the unplanned told lies would have been most difficult to generate (DePaulo et al., 2003; Greene et al., 1985; Walczyk et al., 2009) resulting in the strongest cues for cognitive operations; however, this assumed that participants would use great effort to create a lie without pre-planning. According to Leins, Fisher, and Ross (2013), however, liars may not be working that much harder than truth tellers because one of the main strategies liars use is to recycle true stories. So, similar to telling a preplanned lie that is simply retrieved from memory and hence less cognitively demanding, recycling a true story would not be particularly cognitively demanding and would not result in a strong memory for cognitive operations. If the lie event was already stored in memory, there would be no additional cognitive resources devoted to creating unplanned lies. This could explain why there was no additional benefit of planning the lies in advance in comparison to telling them on the spot. The strategy used for creating the lie determines how cognitively demanding lie telling is and this variability could affect memory for cognitive operations across participants. Polage (2004) showed wide variability in belief in the lie, in that some decreased their belief in the lie and some came to fully believe in it. It was assumed that the source monitoring decision would benefit from the effort involved in telling a lie, however, some lies told might require extreme effort to create while others require less effort than remembering an old true memory. In fact, Memon et al. (2010) found an increase in cognitive operations when participants were telling the truth, so telling the truth can also be cognitively demanding, sometimes more so than lying. In addition, Verschuere, Spruyt, Meijer, and Otgaar (2011) found that lying was less demanding when liars lied more frequently, and that the lying response became more dominant with repeated use. So, lying may be easier for some participants than others. Polage (2012) found that those who lied more often were more likely to show fabrication inflation. As lying increases, the process of lying might get easier, using less cognitive resources to create lies. Also, proficient liars might simply have more preplanned lies available in memory. In the absence of memory for the cognitive demands of lying, the other aspects of telling lies that decrease source monitoring ability may have caused participants to increase their belief in the lies.

Polage (2012) found that those who felt more guilt lying, lied more often and were more likely to believe the lies. These results seem contradictory as you would expect those who feel more guilty lying to do so less often, however, results on cognitive dissonance, suggest that those who feel guilty about lying but do so often are likely to experience psychological discomfort or cognitive dissonance (Festinger, 1957). Believing they didn’t lie is one strategy that can decrease cognitive dissonance and motivated forgetting of having lied or denying information that might go against their preferred reality is one way to bring their beliefs in line with their actions. Shu et al. (2011) and Kouchaki and Gino (2016) showed that cognitive dissonance can result in forgetting of unethical actions. It is possible that participants in this study were faced with whether to believe in an event they have already told someone else was true and of which they have detailed memory. Motivated forgetting could impair memories that contradicted having lied and they may find themselves less certain of the truth. Since motivated liars should maintain consistency and stick with the version of the story that was first made public (Wade et al., 2010) they may increase their doubt in the truth. The change in belief for lies that were told was on average 1.27 points on an 8 point scale which suggests that fabrication inflation may cause a slow eroding of belief, which over time might continue to increase if the lie is maintained and reinforced by others.

Finally, one cannot discount that verbal lying has the memorial benefits of speaking out loud which has been shown to improve memory (Hopkins & Edwards, 1972). It is also possible that the liars were maintaining the lie, but that no memory change actually occurred. When studying deception this is always a concern as we don’t know if participants are believing the lie, trying to dupe the researcher, or even responding to perceived demand characteristics. These are possibilities that cannot be overlooked.

In summary, the current results showed that telling a lie, in contrast to simply planning a lie, resulted in fabrication inflation and led liars to increase their belief in lies told with the intention of deceiving. Based on previous literature, I suggest that the simple, plausible event details that seem familiar and true might increase in belief in the lie. Although one might remember the cognitive effort used to create the memory and reject it as false, it is also possible that proficient liars or those who use recycled versions of the truth might not remember creating a lie. It is also possible that the desire to reduce cognitive dissonance by using lax criterion and motivated forgetting of information that contradicts public “truth”, combined with the negative affect of lying can reduce the liar’s ability or desire to effectively source monitor.

As this is the first study that directly compares telling versus planning a lie, it raises many questions that need to be addressed in future research. The results support the idea that telling a lie results in fabrication inflation but it does not answer the question of why the effect occurs. For example, are participants motivated to remember the content of the lie in order to remain consistent? If motivation to believe affects memory for the truth, one would expect the lies told to be similar over time. Do participants change their “told” lies to be more realistic and hence more believable than their planned only lies (to others and potentially to themselves)? Future research should examine the level of detail provided in the lie stories to determine whether liars do attempt to “keep it simple” as Vrij et al. (2010) suggests and whether the level of detail has an effect on memory. The interview sessions in the current study were not video-recorded and were therefore not able to be analyzed for content. Future research might attempt to examine the consistency of the stories and rate them on Criteria Based Content Analysis (CBCA; Steller & Koehnken, 1989) elements. It is possible that stories that score higher on CBCA may result in more memory distortion.

The effects of lying on belief have been relatively unexplored and the current study suggests that the intentionally deceptive component of lying, not just the creation of the lie, affects belief in the lie. These results suggest that belief change may occur as a result of a deliberate lie and that liars become less confident in the truth after lying. Given that the average person lies at least once a day (Serota & Levine, 2015), the effects of lying on false beliefs have repercussions that affect everyone and continued research into related variables and their effects should be conducted. Although the lies told in this study were low stakes lies, it is possible that the factors associated with lying that might increase source monitoring errors such as discomfort and motivated forgetting would be even stronger in high stakes lies such as those involving perjury and coerced false confessions. This research therefore has applications both to everyday experiences and psychology and law topics.

## Appendix: Questions and Participant Instructions That Were Used During the Written and Oral Interviews

**Table:**

Event	Participant instructions
	Did this really happen to you?
Did you fall off your bicycle?	Yes
Did you shake hands with the president?	No
***Lie 1*** (**prepared and told**- used in written and oral interviews)	Yes
Did you get a hook stuck in your hand while fishing?	No
Did you build a fort?	No
***Lie 2*** (not prepared but **told**- oral)/ ***Lie 3*** (**prepared** but not told- written)	Yes
Did you swallow chewing gum?	Yes
Did you break a favorite toy?	No
***Lie 4*** (**control**- neither prepared nor told)	---

*Note.* The participants were asked to follow the instructions *whether* the event had or had not actually happened to them. Lies 1-4 were based on their responses to the LEI 1. The other events were filler questions given to all participants.
